# Induction of the acid inducible *lipF* promoter is reversibly inhibited in pH ranges of pH 4.2-4.0

**DOI:** 10.1186/s13104-018-3370-1

**Published:** 2018-05-08

**Authors:** Rachelle Torres, Donna Dorriz, Beatrice Saviola

**Affiliations:** 1 Basic Medical Sciences, College of Osteopathic Medicine, Western University of Health Sciences, 309 E. Second St., Pomona, CA 91766 USA; 2 Mercy Medical Center Merced Hospital, 333 Mercy Avenue, Merced, CA 95340 USA

**Keywords:** Acidic stress, *lipF*, Lipase, *Mycobacterium tuberculosis*

## Abstract

**Objective:**

In the human body pathogenic mycobacteria encounter low pH within the phagosomes of macrophages where they reside after being internalized by the host cell. Low pH within macrophages has been shown to induce expression of a variety of genes within these bacteria. It had been previously observed that the *Mycobacterium tuberculosis lipF* promoter is transcriptionally upregulated between pHs 4.5–6.4 in *Mycobacterium smegmatis*, with an upper pH limit of 6.4 capable of promoter induction. To better understand the parameters of acid induced gene expression, we sought to determine the lower pH limit capable of *lipF* promoter induction.

**Results:**

As we had already determined an upper pH limit, we determine here that there is a lower limit of pH’s capable of upregulating the *lipF* promoter, with pH below 4.3 not positively upregulating the promoter. At non-inducing pH 4.2 the bacterial cells remain viable in the absence of acid induced *lipF* promoter upregulation and subsequent exposure to acid pH 5.0 results in *lipF* promoter upregulation. There appears to be a lower limit of pH capable of upregulating *lipF* promoter expression and this limit is not due to cell death.

## Introduction

Tuberculosis continues to be an important health problem in the world with a large fraction of the population infected with *Mycobacterium tuberculosis*, the causative agent of tuberculosis [1]. Like other mycobacteria, *M. tuberculosis* senses and responds to acidic stress [2, 3]. Previously we had identified a 477 base pair region upstream of the *M. tuberculosis lipF* gene that was transcriptionally upregulated by acidic stress [4]. A homologue of the *lipF* gene was previously identified in *Mycobacterium smegmatis* and is implicated in acidic stress [5]. Importantly, a transposon insertion mutant in between the *lipF* gene and its 59 base pair acid inducible minimal promoter resulted in a mutant *M. tuberculosis* that does not produce LipF and was more attenuated in mice and macrophages indicating the gene product aids *M. tuberculosis* in resisting acidic stress [6]. The product of the *lipF* gene is predicted to be a lipase or esterase, and proven to act as a lipase [7].

The *lipF* promoter region was reduced to a 59 base pair minimal region which retained the ability to be upregulated by acidic stress [8]. Within this region we also identified a − 10 six base pair region proposed to bind RNA polymerase and analyzed it by mutational analysis [9]. As the original pH at which the *lipF* promoter had been identified was pH 4.5, the highest pH to also induce the promoter was sought and determined to be pH 6.4 [10]. This is a pH that may be encountered within the phagosomes of macrophages where *M. tuberculosis* resides within the human body. During infection *M. tuberculosis* is phagocytosed by macrophages and the pH can drop to as low as 4.5 within phagosomes but can then increase to a range from pH 6.0–6.5 [11–14]. Thus the previously described range of *lipF* promoter activity between pH 4.5 and 6.4 is well within this spectrum allowing the bacterium to be more resistant to acidic stress. In this present study we determine a lower pH limit of *lipF* promoter upregulation, which may be important to understand acid sensing mechanisms of mycobacteria.

## Main text

### Results

**Fig. 1:**
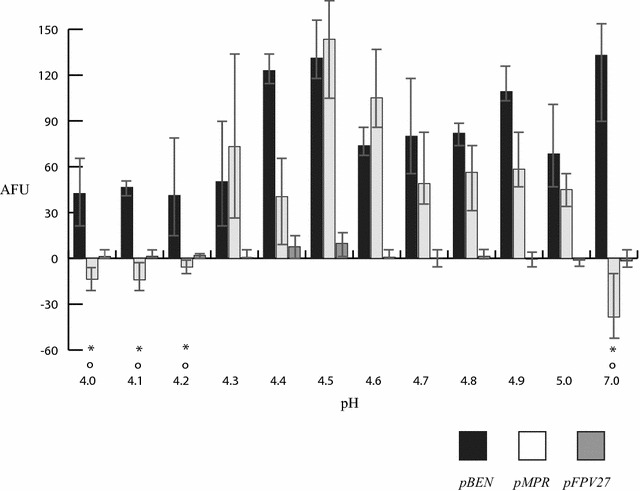
*M. smegmatis* containing *pBEN*, *pMPR*, or *pFPV27* were grown to mid-logarithmic phase in pH 7.0 media and the pH was then shifted to pH 4.0 through 7.0 for a 3 h exposure. Fluorescence and OD_600_ were measured. AFU is adjusted fluorescence units and calculated by fluorescence units/optical density units at 600 nm. The data are presented as the mean ± the standard deviation. *P < 0.03, significantly different from pH 4.5; ^o^*P* < 0.04, significantly different from pH 4.3. There was no statistical difference of cells bearing *pMPR* at pH 4.4, 4.5, 4.6, 4.7, 4.8, 4.9, and 5.0 compared to pH 4.3. Statistical difference was defined as a *P* < 0.05. The sample size was 3 and represents biological replicates

**Fig. 2:**
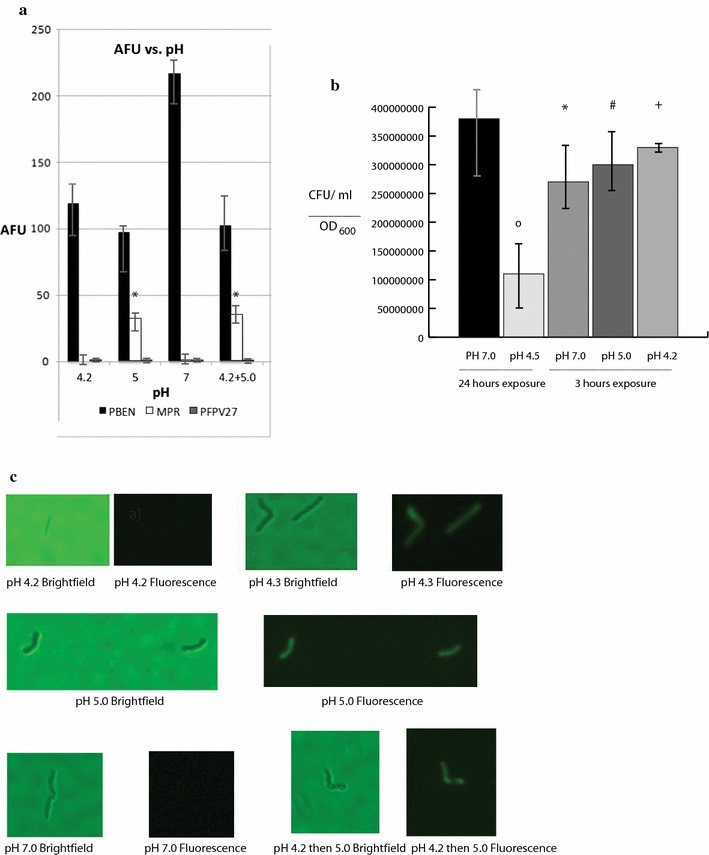
**a** To test if induction at pH 4.2 is reversible, *M. smegmatis* containing *pBEN*, *pMPR,* or *pFPV27* were grown to mid-logarithmic phase at pH 7.0, then exposed to pH 4.2, 5.0, or 7.0 for 3 h. An additional culture had been previously grown at pH 7.0 to mid-logarithmic phase, shifted to pH 4.2 for 3 h, and then shifted to pH 5.0 for an addition 3 h. AFUs were determined as previously described. The data are presented as the mean ± the standard deviation. *P < 0.04, significantly different from pH 4.2 3 h of exposure. The sample size was 3 and represents biological replicates. **b** To determined viability of mycobacteria exposed to acidity, the *M. smegmatis* containing *pMPR* were exposed to pH 7.0, 5.0, or 4.2, diluted to 1:10^5^, and plated onto 7H10 plates. Bacterial colony forming units (CFU)/ml of the undiluted sample were quantitated from the bacterial plates and were normalized by dividing by the OD_600_ of the bacterial undiluted samples. Exposure to 3 h of acidity did not reduce mycobacterial viability in any of the conditions tested including 3 h of exposure to pH 4.2. As a control *M. smegmatis* was grown to mid-logarithmic phase and then exposed to pH 4.5 or 7.0 for 24 h. The bacteria were then diluted and plated onto agar plates and the CFU/ml normalized for OD_600_ as before. As expected exposure to 24 h at pH 4.5 resulted in a 75% reduction of viability of the mycobacteria whereas exposure to pH 7.0 did not. The data are presented as the mean ± the standard deviation. *P < 0.05, significantly different from pH 4.5 24 h exposure; ^#^*P* < 0.03, significantly different from pH 4.5 24 h exposure; ^+^*P*  < 0.02, significantly different from pH 4.5 24 h exposure; ^o^*P* < 0.02, significantly different from pH 7.0 24 h exposure. There was no statistical difference for pH 4.2 or 5.0 3 h of exposure compared to pH 7.0 3 h of exposure. There was also no statistical difference between pH 4.2, 5.0, or 7.0 3 h of exposure compared to pH 7.0 24 h of exposure. Statistical difference was defined as a *P* < 0.05, the sample size was 3 and represents biological replicates. **c** *M. smegmatis* containing *pMPR* was grown to mid-logarithmic phase and exposed to pH 4.2, 4.3, 5.0, or 7.0 for 3 h, or 4.2 3 h and then 5.0 3 h. Individual *M. smegmatis* bacilli were visualized via differential interference contrast microscopy (DICM) and fluorescence microscopy at ×40 magnification. This experiment was repeated three times with similar results. Approximately 7 images were sampled for each condition

The minimal acid inducible *lipF* promoter of *M. smegmatis* is composed of 59 base pairs upstream of the *M. tuberculosis lipF* gene and this was fused to *gfp* to create the plasmid *pMPR* (Table 1) [8]. This promoter is upregulated by acidic stress within *M. tuberculosis* as well as within *M. smegmatis*, consequently an acid sensing apparatus is similar in these species [4]. As we had previously identified the highest pH capable of inducing the *lipF* minimal acid inducible promoter to be pH 6.4, here we test the lowest possible pH that can induce the *lipF* promoter fused to *gfp* (*pMPR*). We found that all pHs tested except for pH 4.0, 4.1, and 4.2 could induce the *lipF* promoter with maximal induction being at pH 4.5 (Fig. 1). This maximum induction coincides with the original pH used to identify the *lipF* promoter’s acid induction [4, 10]. *M. smegmatis* containing *pFPV27* with a promoterless *gfp* (Table 1) had little green fluorescent protein (GFP) production. Negative values for *M. smegmatis* containing *pMPR* indicate the cells produce less GFP than the bacteria containing promoterless *pFPV27*. *M. smegmatis* containing the *pBEN* plasmid which produces GFP constitutively from a heat shock promoter (Table 1) had substantial GFP production as indicated (Fig. 1). As we expected that pH 4.2 would be lethal to mycobacteria and would explain a lack of *lipF* induction, we tested mycobacterial killing at this pH, and at the same time assayed *lipF* induction. As before, acid induction of the *lipF* promoter occurred at pH 5.0 and not at pH 4.2 or pH 7.0 (Fig. 2a). In addition no acid induced bacterial killing occurred at pHs 4.2, 5.0, or 7.0 with 3 h of exposure, consequently cell death does not explain the lack of induction (Fig. 2b). Exposure to 24 h of acid media at pH 4.5 resulted in 75% reduction in viability compared to exposure to pH 7.0 media (Fig. 2b). To further investigate we repeated induction at pH 4.2 for 3 h, centrifuged the bacterial cells, washed them, and resuspended them in pH 5.0 for another 3 h and observed positive *lipF* induction (Fig. 2a). The lack of induction of the *lipF* promoter is reversible, as removal of pH 4.2, and exposure to pH 5.0 resulted in *lipF* promoter induction. Fluorescence photography was performed on *M. smegmatis* at pH 4.2, 4.3, 5.0, 7.0 3 h, or 4.2 exposed for 3 h, centrifuged, washed, and induced at pH 5.0. *M. smegmatis* with an *mpr*-*gfp* fusion fluoresces brightly after exposure to pH 5.0 and 4.3, but no fluorescence after exposure to pH 7.0 or pH 4.2 (Fig. 2c). Strikingly fluorescence was also observed after bacterial exposure to pH 4.2 for 3 h, washing, and exposure to pH 5.0 for an additional 3 h (Fig. 2c). This fluorescence was observed at the end of the 3 h incubation at pH 5.0.

**Table 1:** Shuttle plasmids used in the experiments that can be amplified in *E. coli* and transformed into *M. smegmatis*

*pFPV27*	Reporter plasmid with promoterless *gfp*
*pBEN*	Reporter plasmid with constitutive heat shock promoter driving constitutive expression of *gfp*
*pMPR*	Reporter plasmid with *lipF* minimal acid inducible promoter driving expression of *gfp*

### Discussion

In this study we show a lower limit where acidity no longer induces the *lipF* promoter in *M. smegmatis*. In vivo, *M. tuberculosis* is taken up by macrophages into phagosomes during the infectious process. When macrophages are induced with Interferon gamma (INF-γ), pH inside the phagosomes decreases to 4.5 and rebounds to pH 6.0 or above (11, 12, 13, and 14). The *lipF* promoter would be active during these in vivo conditions. In this study we determined the acidic pH at which *lipF* fails to be upregulated is pH 4.2 and this pH is not reported to be present within phagosomes of macrophages that contain viable *M. tuberculosis* bacilli. The *lipF* promoter may have evolved to detect pHs actually present within phagosomes.

The transcription factor PhoP has been shown to bind within the *lipF* promoter but it is unclear if it responds to external acid stress [15]. PhoP is part of a two component system which employs a membrane bound sensor kinase to sense external stimuli and in response phosphorylates PhoP, though acid stress is not known to be involved in PhoP phosphorylation [15]. Sigma factor binding site analysis revealed that the *lipF* promoter likely uses a principle sigma factor and a deletion strain of the *sigF* gene encoding a stress response sigma factor revealed that it is likely not involved in *lipF* promoter regulation as the promoter is upregulated in this strain [4, 9]. It remains unclear the exact nature of the acid sensing mechanism and the exact transcription factors involved in *lipF* promoter upregulation.

Bacterial killing or toxicity is unlikely to be the origin of the lack of ability to upregulate the *lipF* promoter at pH 4.2 as bacteria were measured to not lose viability in 3 h acid exposure time frame. In the *lipF* promoter upregulation system a protein factor present on the mycobacterial cell surface may be responsible for sensing pH with an optimum function between pH 6.4 and 4.3. At pH 4.2 this factor may be reversibly in an inactive configuration to signal to the *lipF* promoter within the cell. Upon washing away pH 4.2, and resuspending in pH 5.0 the new environment is capable of converting the conformation to an activating form. In vivo pH 4.2 is likely never experienced within phagosomes and the factor evolved to be optimally active at acidic pHs encountered. With manipulation of the intraphagosomal pH to be slightly lower than normal (pH 4.5) to 4.2 or lower, genes normally thought to be active during exposure to an acidic phagosome may not be upregulated and decrease *M. tuberculosis* adaptability and increase bacterial cell death.

### Conclusions

Here we show the lower pH limit for *lipF* promoter induction is pH 4.3. Likely the failure to upregulate the acid induced *lipF* promoter from pH 4.2-4.0 results from dysregulation of the acid sensing/promoter induction machinery that is not related to mycobacterial cell death. This machinery and process may be manipulated in the future using pharmacotherapy in *M. tuberculosis* infected individuals to lower intraphagosomal pH below the pH 4.3 limit of acid induction.

### Methods

#### Strains and media

*Escherichia coli* strain DH5α (ATCC 67878) was used in all experiments to generate and amplify plasmid constructs for further use. *E. coli* was grown in Luria–Bertani broth and on Luria–Bertani agar petri plates (Fisher Scientific, BP1427-500 and DF0445-17-4). *M. smegmatis* Mc^2^ 155 (ATCC 700084) was grown at 37 °C in Middlebrook 7H9 broth (Becton–Dickinson, DF0713-17-9) supplemented with 10% ADC (bovine serum albumin-Fisher Scientific BP1600-100, dextrose-EMD DX0145-1, and NaCl-EMD SX0420-1), 0.025% Tween 80 (polyoxyethylenesorbitan monooleate—Fisher Scientific T164-500), and 2% glycerol (Hoefer GR124-1) in rolling liquid media culture, and in Middlebrook 7H10 agar (Becton–Dickinson, DF0627-17-4) with 10% ADC for solid surface growth. *E. coli* was transformed with 50 ng of *pFPV27*, *pMPR*, and *pBen*, (Table 1), containing promoterless *gfp* [16–18], the 59 base pair minimal acid inducible *lipF* promoter fused to *gfp* [8], and the heat shock promoter fused to *gfp* respectively [19]. 25 μg/ml kanamycin (Fisher Scientific AAJ6066803) was utilized for selecting resistant plasmids. Plasmids were isolated using Wizard minipreps (Promega, PR-A1330). 50 ng of purified plasmids were transformed via electroporation into *M. smegmatis* and selection occurred on 7H10 agar with 10% ADC and 25 μg/ml kanamycin.

#### Acid promoter induction

To create acidic media, 2% HCL (EMD Millipore, MHX06074) was added to 20 ml of 7H9/ADC broth dropwise to reach the desired pH between 5.0 and 4.0. *M. smegmatis* containing plasmids were grown in pH 7.0 Middlebrook 7H9 broth to an optical density at 600 nm (OD_600_) on a Genesys10uv spectrophotometer (Thermofisher 840-208100) of 0.5 (logarithmic phase), the cells were centrifuged for 2 min at 12,000 rotations per minute (RPM) and 24 °C in a microcentrifuge (BioRad Model 16K—1660602EDU), supernatant removed, and cells resuspended in 7H9 culture media (Becton–Dickinson) at pHs 5.0-4.0, or neutral pH 7.0, and grown for an additional 3 h. 3 h was chosen as this is the approximate generation time of the mycobacteria. All promoter inductions were performed at 37 °C, and all pH points were tested in triplicate. To terminate inductions, all samples were vortexed with 4 mm glass beads (Fisher, 50-872-931) to eliminate clumping and were diluted to the same optical density at 600 nm in 7H9 broth. Fluorescence of the samples was measured on a TD-700 Turner designs fluorometer (7000-998—Turner Designs) with a 486 nm (nanometer) excitation filter and a 510–700 nm emission filter. Adjusted fluorescence units were determined to be fluorescence units/optical density units at 600 nm. All measurements in the figures are mean of the values and the error bars are the standard deviation. To further investigate non-inducing pH 4.2, *M. smegmatis* was grown in 7H9 pH 7.0 until reaching OD_600_ of 0.5, exposed to 7H9 media at pH 4.2 for 3 h, centrifuged, washed, and then resuspended in pH 5.0 for another 3 h, and measured on a TD-700 Turner designs fluorometer as previously described. pH points were tested in triplicate.

#### Microscopy

Bacteria were visualized via differential interference microscopy, and also fluorescence microscopy using a Nikon Eclipse E600. The same field of slide was captured by differential interference contrast microscopy and by fluorescence at 40× magnification to record identical *M. smegmatis* cells.

#### Determination of cell viability

*Mycobacterium smegmatis* was grown to mid-logarithmic phase, exposed to pH 7.0, 5.0 or 4.2 for 3 h, or to pH 7.0 or 4.5 for 24 h. Cells were vortexed with 4 mm glass beads (Fisher) and the optical density at 600 nm was determined. To plate the bacteria, the samples were diluted 1:10^5^, and 1:10^6^ and 100 μl of samples were plated onto 7H10 plates. *M. smegmatis* was incubated at 37 °C for 3 days and the number of colonies was counted. The number of bacterial colony forming units (cfus)/ml for an OD_600_ of 1 was determined. All pH points were tested in triplicate and the data was described as the mean of the values and the error bars are the standard deviations.

#### Statistical analysis

Three biological replicates were performed for each condition and data are represented as the mean ± standard deviation. An unpaired t test was used to compare means and statistical significance was defined as *P* < 0.05.

## Limitations

This data was obtained from *M. smegmatis* cultures in vitro and may need to be repeated in *M. tuberculosis.* Only pHs to pH 4.0 were tested and sample pHs below this limit could be investigated in the future.

## Abbreviations

AFU: adjusted fluorescence units
CFU: colony forming units
*E. coli*: *Escherichia coli*
GFP: green fluorescent protein
INF-γ: interferon gamma
*M. smegmatis*: *Mycobacterium smegmatis*
*M. tuberculosis*: *Mycobacterium tuberculosis*
nm: nanometer
OD_600_: optical density at 600 nm
RPM: rotations per minute
